# Society, organizations and the brain: building toward a unified cognitive neuroscience perspective

**DOI:** 10.3389/fnhum.2015.00289

**Published:** 2015-05-19

**Authors:** Carl Senior, Nick Lee, Sven Braeutigam

**Affiliations:** ^1^School of Life & Health Sciences, Aston UniversityBirmingham, UK; ^2^School of Business and Economics, Loughborough UniversityLoughborough, UK; ^3^Oxford Centre for Human Brain Activity, Oxford UniversityOxford, UK

**Keywords:** organizational cognitive neuroscience, functional brain imaging, neuromarketing, neuroeconomics

The Oxford English Dictionary contains the following entry for the word “postal” as:

• adjective relating to or carried out by post.
  – PHRASES go postal US informal go mad, especially from stress. With reference to cases in which postal employees have run amok and shot colleagues.

Even a superficial knowledge of recent events may lead to the conclusion that the contemporary organization is perhaps not an easy thing to manage in a way that guarantees both economic and social prosperity. As such, it seems to be part of the modern human condition to be at least somewhat unhappy, stressed, or otherwise negatively impacted by either organizational life itself, or the impact of organizations on today's society. Fortunately, however, worst-case scenarios—as implied by the OED above—are very rare.

It does not come as a surprise then, that researchers have expended considerable efforts on exploring and understanding the formation, management, and ethical sustentation of organizations of all kinds and sizes, from bleeding-edge venture enterprises operating in break-neck markets to perhaps non-competitive, non-profit charities. Drawing from an interest in the negative effects workplaces can have on individuals, some of us published a clarion call, raising questions about how a better understanding of our biological systems could inform an understanding of the social behavior that we manifest within organizations (Butler and Senior, 2007a,b). The critical question here is how the organization and the individual interact and influence each other, given that it that organizations are designed as they are by the very same species which will work in them, and equally important how cognitive neuroscience in particular can help to unravel such mechanisms.

Scholars have indeed begun to explore the neuroscience of organizational behavior. These efforts go under the names of Organizational Neuroscience and Organizational Cognitive Neuroscience, terms that refer to cross-disciplinary perspectives on organizational research, which take as their foci of study the cognitive mechanisms that drive human behaviors in response to organizational manifestations (Senior et al., 2008, 2011; Becker et al., 2011; Lee et al., 2012a). Such approaches seem to have some merit in the study of the effects of organizational life on human beings, and also on how one can mitigate the more deleterious effects that appear inherent to such contexts. However, even with such rich empirical intercourse there remains an opportunity to examine further the current state of the art research endeavors that span the biological and organizational domains to inform our understanding of the type of social behavior that most of us will carry out most days for most of our lives.

The articles contained within this research topic do just that, and go beyond merely explicating further the possible mechanisms that drive “social behavior that occurs within organizational manifestations” (Senior et al., 2011, p. 2) but ensure that such an understanding actually informs our knowledge of a socially relevant and species specific social behavior. In the call for papers we chose not to restrict the nature of articles, but to ensure that all submissions could inform our wider understanding social behavior in this applied context (Waldman, 2013). The resulting submissions can be loosely grouped into four main clusters–(a) general management, (b) leadership, (c) neuromarketing science, and (d) papers that have made specific recommendations for subsequent work.

To fully realize the potential for the impact of these articles, it is important to first reflect upon the industrial revolution and how it showed that complex products could most profitably be made by breaking them up into small specialized, repetitive tasks. As far back as the early 20th Century, with the emergence of “scientific management” (e.g., Taylor, 1911), and the principles of Fordism, the place of humans in this workflow was also treated as a mechanistic process, to be designed in such a way as to maximize efficiency and minimize defects. In such a context, one could be forgiven for wondering whether working in such organizations was what humans were ideally suited to. Even so, it is undeniable that humans are the only species to have organized itself into abstract organizations (i.e., not solely related to survival or socialization), suggesting that perhaps something about this ability does confer a collective advantage, if not an individual one. In such a context, one would be forgiven for fearing that the application of cognitive neuroscientific technology to helping us understand more about our behaviors within the workplace may drive the onset of what might become a neo-scientific management; one that sees the data from workers as merely a mechanism to maximize efficiency and minimize defects. Yet the articles contained in this research topic show that this is far from the case and, rather than driving biological reductionism, the articles collectively demonstrate the significant impact that such approaches can bring to helping us understand human behavior.

In a novel approach to addressing a significant question, Block et al. (2014) carried out a large-scale interrogation of an existing database on media behavior and found a significant relationship between media usage e.g., Internet, television and other social media, and self-reports of depression. Christoff (2014) takes exploration of the relationship between organizational settings and mental-health a stage further, and argues that a discourse exploring the role emotions play in organizational decision-making is needed. In light of the fact that in modern organizations, so many of us place such heavy emphasis on such media outlets when enacting our working roles, considering the possible effect that they may have on mental health ensures that we consider the welfare of the individual workers of paramount importance (see also Senior and Lee, 2013 for further discussion here).

Taken together, the work by Spain and Harms (2014) and Verbeke et al. (2014), converges on a greater understanding of individual behavior within an organization at a genetic level. This socio-economic approach is then examined further with the submission by Foxall (2014a), who suggests a model for effective managerial behavior; that is, the function of competitive neural systems. The notion of dual systems operating in competition to drive effective managerial behavior was examined further with work by Boyatzis et al. (2014) who carried out an fMRI study identifying antagonistic neural systems responsible for different types of leadership behaviors.

Such work continues to inform our understanding of how social cognitive neuroscience (Ochsner and Lieberman, 2001) can advance organizational research—a project essentially started by our earlier work (e.g., Lee et al., 2012a). In particular, and possibly as the result of serendipitous collaboration, neuroscientific measuring tools such as functional magnetic resonance imaging (fMRI) and magnetoencephalography (MEG) have been applied to a number of organizational research questions (e.g., McClure et al., 2004; Braeutigam, 2005; Deppe et al., 2005). Such approaches have given the world terms as “neuroeconomics” (Braeutigam, 2005) and “neuromarketing” (Breiter et al., 2015b), and have inspired some considerable controversy in the scientific press (e.g., Nature Neuroscience July 2004). Such debate is healthy and as is shown by Butler (2014), Lindebaum (2014), and Waldman (2013) helps to drive consolidation of theory and clarification of approaches.

This, then, is the foundation of Organizational Cognitive Neuroscience (OCN), which as an approach brings together diversity in research approaches that use neuroscientific theories and methods to examine organizational research issues (Senior et al., 2011; Lee et al., 2012a). Indeed, the benefits of an OCN approach are exemplified by Foxall (2014b), Walla et al. (2014) and Breiter et al. (2015a), who each describe how the study of exchanges within a market scenario can provide insights more general human behavior, which in turn would lead to a more “integrated science of influence” (Breiter et al., 2015b p. 1). These scholars highlight both theoretical and methodological advances within mainstream cognitive neurosciences and the implications for a greater understanding of human behavior when market exchanges are specifically investigated. Such methodological advances are explored further with work by Kopton and Kenning (2014) and Burgess (2013) who, among other things, develop novel statistical approaches for the analysis of hyper scanning data—which looks likely to be a crucial technique in exploring the sort of interactions so central to organizational life.

That said, such work clearly shows that theoretical advancement is not dependent on simply grafting advanced measurement tools (such as fMRI) on to existing theories, as implied by many early uses terms such as “neuromarketing” (and here we recognize that Breiter et al. (2015b) clearly define a more scientifically-rigorous useage of neuromarketing). Instead, the OCN approach explicitly recognizes that it is the interaction between cognitive neuroscience and organizational research as distinct fields of research which is critical—incorporating not just new methods, but also new theoretical explanations. In such a way, the field can lead to advances in both its parent disciplines (Lee et al., 2012b).

We have previously conceptualized OCN as an approach that considers human behavior made in response to organizational manifestations (e.g., products, advertisements) as a set of theoretical layers, each building upon the last to add more context-specific theory (Lee et al., 2012b). At the most abstract level, the behavior of individuals and groups at the intersection between the organization and the human is considered. Yet such behavior is a subset of human social behavior in general. Therefore it is an additional layer of theory that can be added upon social psychology. In turn, social psychology is founded on theories of cognitive psychology, which also impact directly on many of our responses to organizational manifestations such as advertisements and products. At an even more basic level, are the lower-level brain systems and structures that drive such cognitions, analysis here could be termed the neural level of analysis. To facilitate investigations across the various layers of analysis that are diagnostic of the organizational cognitive neuroscience approach, Rippon et al. (2014) provide a set of recommendations that could be adopted when studying the effects of gender on particular task.

The organizational, social, and neural levels that are described above have been the focus of existing OCN theory (e.g., Lee and Chamberlain, 2007). Yet, at a more fundamental level one can also describe the adaptive forces that have shaped our brain physiology in an evolutionary advantageous manner (Saad and Greengross, 2014). Knowledge of the evolutionary adaptations that may mediate our behavior at the social and ultimately organizational level is essential to complete the explanation of why we behave in the way we do, and also critical in understanding the potential negative (and positive) influence of organizational life on human beings.

To move back to the example of “scientific management” previously alluded to; an understanding of whether the ability to focus on repetitive small tasks may have conferred an evolutionary advantage in the past (which therefore would have led to a predilection for this ability in humans) may then lead to greater understanding of whether scientific management principles are likely to be beneficial to employees. Importantly, this is quite apart from the logical principles of the approach, which may indeed suggest that it may be the most efficient manner with which to produce a complex product with minimum defects. Indeed, the key social processes (within organizations) that humans have a predilection toward are discussed subsequently.

Such an idea has been developed further with the work by Saad and Greengross (2014), who go so far as to say that an understanding of evolutionary theory is of paramount importance when using cognitive neuroscientific technology to explore organizationally-relevant behaviors. However, it is with the work by Spisak et al. (2014) and Price and Van Vugt (2014) where the importance of studying adaptive behaviors and the role that they may play in facilitating effective organizational is made crystal clear (See also von Rueden, 2014). Developing this further, Susac and Braeutigam (2014) describe how an understanding of the neural substrates underpinning mathematical cognition may in fact facilitate the ability for mathematical reasoning—which itself has implications for the subsequent design of effective education.

Here it is clear that it is not possible to fully understand a given organizationally-relevant behavior by ignoring the various interweaved layers of theory introduced above, Focusing on the neural level—without taking into account the more fundamental evolutionary level, or even the more abstract organizational and social levels—is likely to result in important explanatory contextual factors being overlooked. OCN explicitly recognizes the symbiotic relationship between the layers of theory and in doing so develops more rigorous testable hypotheses, and ties this to advances in research methods that can more accurately test these hypotheses. The studies noted above develop existing OCN theory (e.g., Butler and Senior, 2007a) to show in more depth the evolutionary processes that may impact on our organizationally-relevant actions. The focus here is on how the neural and evolutionary levels interact, and the question of whether such adaptations actually can influence our behavior within, and our response to, organizations and their manifestations.

As noted above, organizations that are designed around the social processes that humans have a predilection for are likely to operate more efficiently. Yet we should not consider the application of neuroscience to understanding organizational behavior as a means merely to make such organizations more efficient. In spite of the working environment being constantly in flux, the central concept of organizational behavior has and will always remain the same. Most of us are likely to spend a major proportion of our lives in a work-related environment. One may argue thus that organizational cognitive neuroscience is an approach by which to understand the cognitive signature of our own species-specific social behavior.

We would like to dedicate this research topic to the many reviewers who considered the submitted papers in such a timely fashion–without them this collection would not have happened.

## Conflict of interest statement

The authors declare that the research was conducted in the absence of any commercial or financial relationships that could be construed as a potential conflict of interest.
