# Corrigendum: Society, Organizations and the Brain: Building toward a Unified Cognitive Neuroscience Perspective

**DOI:** 10.3389/fnhum.2015.00411

**Published:** 2015-07-20

**Authors:** Carl Senior, Nick Lee, Sven Braeutigam

**Affiliations:** ^1^School of Life and Health Sciences, Aston University, Birmingham, UK; ^2^School of Business and Economics, Loughborough University, Loughborough, UK; ^3^Oxford Centre for Human Brain Activity, Oxford University, Oxford, UK

**Keywords:** organizational cognitive neuroscience, loss aversion, fMRI analysis, society, cognitive neuroscience

In the above editorial the following reference:

Breiter, H. C., Viswanathan, V., Lee, S., Gilman, J. M., Kim, B. W., Lee, N., et al. (2015a). Age-related striatal BOLD changes without changes in behavioral loss aversion. *Front. Hum. Neurosci*. **9**:176. doi:10.3389/fnhum.2015.00176

Was incorrectly cited and should have been reported as:

Viswanathan, V., Lee, S., Gilman, J. M., Kim, B. W., Lee, N., Chamberlain, L., et al. (2015). Age-related striatal BOLD changes without changes in behavioral loss aversion. *Front. Hum. Neurosci*. **9**:176. doi:10.3389/fnhum.2015.00176

Accordingly, the citation Breiter et al. (2015a) in paragraph 10 should read Viswanathan et al. (2015), and, as a consequence, the citation (same paragraph) Breiter et al. (2015b) should read Breiter et al. (2015). This error occurred due to the fact that an outdated author list on this respective citation was originally processed and this Corrigendum serves to correct this.

## Conflict of Interest Statement

The authors declare that the research was conducted in the absence of any commercial or financial relationships that could be construed as a potential conflict of interest.

